# Cardiovascular Disease in the Indo-Caribbean Population: A Scoping Review

**DOI:** 10.7759/cureus.15375

**Published:** 2021-06-01

**Authors:** Neha Bapatla, Uma D Ramoutar, Natasha Sharma, Anjali Ramoutar, Valentina L Ortega, Anita Goorachan, Farzanna Haffizulla

**Affiliations:** 1 College of Allopathic Medicine, Nova Southeastern University Dr. Kiran C. Patel College of Allopathic Medicine, Davie, USA; 2 Medicine, St. George's University School of Medicine, True Blue, GRD; 3 Epidemiology and Public Health, Nova Southeastern University Dr. Kiran C. Patel College of Allopathic Medicine, Davie, USA; 4 Bioengineering, Rice University, Houston, USA; 5 Public Health, Nova Southeastern University Dr. Kiran C. Patel College of Osteopathic Medicine, Davie, USA; 6 Internal Medicine, Nova Southeastern University, Fort Lauderdale, USA

**Keywords:** indo-caribbean, west indian, caribbean diaspora, caribbean, cardiovascular disease, myocardial infarction, atherosclerosis, hypertension, public health, social determinants of health

## Abstract

At the beginning of the 20th century, there was a shift in disease patterns from that of communicable disease to noncommunicable disease (NCD). As a result, cardiovascular disease (CVD) has emerged as a leading cause of morbidity and mortality worldwide. Its incidence and effect on various populations at a molecular level as well as clinical implications have been heavily studied; however, its role in morbidity and mortality in the Indo-Caribbean population is often overlooked. The Caribbean diaspora is a vibrant and heterogeneous culture, encompassing individuals with ancestries from across the world including the Indian subcontinent and Africa. Abundant research is consistently conducted on these populations, but limited research exists on how the interplay between genetics and environment translates to the manifestation of various diseases in the Indo-Caribbean population. This scoping review aims to identify and assess the current literature within the past 10 years conducted on CVD in Indo-Caribbeans in order to gain a thorough understanding of disease and management to improve health outcomes. Additionally, this review aimed to identify gaps in research that require further study to gain a better understanding of relevant variables affecting disease outcomes in the Indo-Caribbean population. Multiple health databases were queried, and the initial search yielded over 3,000 results. However, after screening with the inclusion and exclusion criteria established, the final search included less than 1% of the papers initially searched. This search yielded data that included treatment and management of myocardial infarction, hypertension, and atherosclerosis, but notably did not yield papers that discussed the relationship between social determinants of health and CVD in Indo-Caribbeans. Florida and New York are prominent states that have robust Indo-Caribbean populations; the lack of research renders these states vulnerable to improving health outcomes in these patients. The authors call for increased focus on this population in research studies and efforts to improve the quality of the data collected through stratification by ethnicity. Robust data may allow for improvement in the treatment and management of CVD in Indo-Caribbeans, which offers a more proactive rather than reactive approach to decreasing morbidity and mortality.

## Introduction and background

The transition to the 20th century is marked by the Epidemiologic Transition during which the global population experienced shifts in patterns of disease, mortality, leading causes of death, and fertility as well as a shift from communicable disease to noncommunicable disease (NCD) [1,2]. Associated with this shift, cardiovascular disease (CVD) has been declared as the leading cause of morbidity and mortality worldwide, with ischemic heart disease (IHD), cerebrovascular disease, hypertensive heart disease, cardiomyopathy, rheumatic heart disease (RHD), and atrial fibrillation accounting for 95% of all CVD-related deaths [1,3,4-6]. CVD was responsible for an estimated 16.7 million deaths globally in 2010; this number has since increased by 12.5% and is projected to exceed 23 million by 2030 [7]. Accounting for approximately one-third of all global deaths, CVD mortality rates are equivalent to the combined number of deaths by infectious disease, nutritional deficiencies, and maternal and perinatal conditions [3,8]. Since 2010, more than 80% of deaths related to CVD have occurred in lower and middle-income countries (LMICs) [9-11]. While risk factors such as tobacco use, hyperlipidemia, and hypertension (HTN) have traditionally been implicated in the increasing incidence of CVD, social determinants of health including poorly structured health systems, lack of health policies, and other barriers to CVD prevention and care have more recently been thought to play an even larger role [1]. Noteworthy challenges to improving CVD morbidity and mortality rates in LMICs include insufficient healthcare spending, poor governance, inefficiencies in care delivery systems, and a predominant focus on reactive care rather than prevention [12].

It is imperative to recognize the immense economic burden of CVD in LMICs. In addition to affecting one at the individual and household levels, disability related to CVD impacts society on institutional and governmental levels [13]. The estimated total economic loss related to CVD between 2011 and 2015 in LMICs amounted to approximately $3.7 trillion, accounting for half of the NCD economic burden and 2% of the gross domestic product across LMICs [14].

In 2013, the World Health Organization responded to the economic burden and increasing mortality rates of NCD by launching the 25x25 Global Action Plan, which aimed to reduce NCD-related premature mortality by 25% by 2025 [15]. In accordance with this plan, sustainable development goals have been established to form the basis for measuring the progress of countries, specifically related to improvement in health outcomes until 2030 [16]. Due to the overwhelming lack of reliable data that estimates CVD burden in a number of regions globally, various organizations have been met with challenges in their attempts to establish prevention and management strategies [15]. Despite many of the undertaken interventions, LMICs may be unable to achieve the 25% target reduction in CVD mortality by 2025 [15]. Even if they are able to meet the universally adopted risk factor targets, barriers in access to healthcare have been predicted to contribute more to the CVD burden than traditional risk factors [12]. In order for the aforementioned actions to be effective at reducing CVD mortality rates, reliable global data that include the impact of clinical risk factors, social determinants of health, and barriers to healthcare and CVD treatment must be collected to design and implement evidence-based policies [16].

The Caribbean, also referred to as the West Indies, is a diverse region consisting of several nations geographically situated on the Caribbean plate between North and South America. Previously colonized by Spain, Britain, the Netherlands, and France, nations in the Caribbean are largely classified as developing nations with gross national income ranging from $800-$30,000 per capita [17]. The rich culture of the Caribbean stems from its heterogeneous post-colonization population. Perhaps due to the diverse contextual, social, and cultural factors, the people of the region face unique epidemiological and health-related challenges. These include access to and trust in healthcare, low levels of health literacy, and a shortage of resources to provide adequate care - challenges which often follow West Indians who immigrate to other nations. These challenges, coupled with those obstacles faced by the general immigrant population, often result in Caribbean immigrants being overlooked in matters related to improving health outcomes. A comprehensive community health assessment conducted among the Indo-Caribbean population of Queens, New York, found that while most respondents reported their health as “good, very good, or excellent,” 65% of respondents had CVD - the top health concern among those that participated in the needs assessment [5]. Results from this study thus evinced the lack of public consciousness among Indo-Caribbeans relating to what constitutes “good” health. Health concerns are further exacerbated by the lack of population data representation relating to this sub-group. For example, the US Census does not include the option to mark Indo-Caribbean as ethnicity, which complicates the ability of epidemiologists and public health officials to obtain vital information about this sizable group. Without disaggregated data, it can be difficult to assess epidemiological patterns and implement appropriate interventions. While literature suggests that CVD disproportionately affects the Indo-Caribbean diaspora, there is little empirical evidence to support this notion [18, 19]. With this understanding, a literature review to assess the number and quality of publications related to CVD in the Indo-Caribbean population is the ideal first step in improving health outcomes in this underrepresented population.

## Review

**Figure 1:**
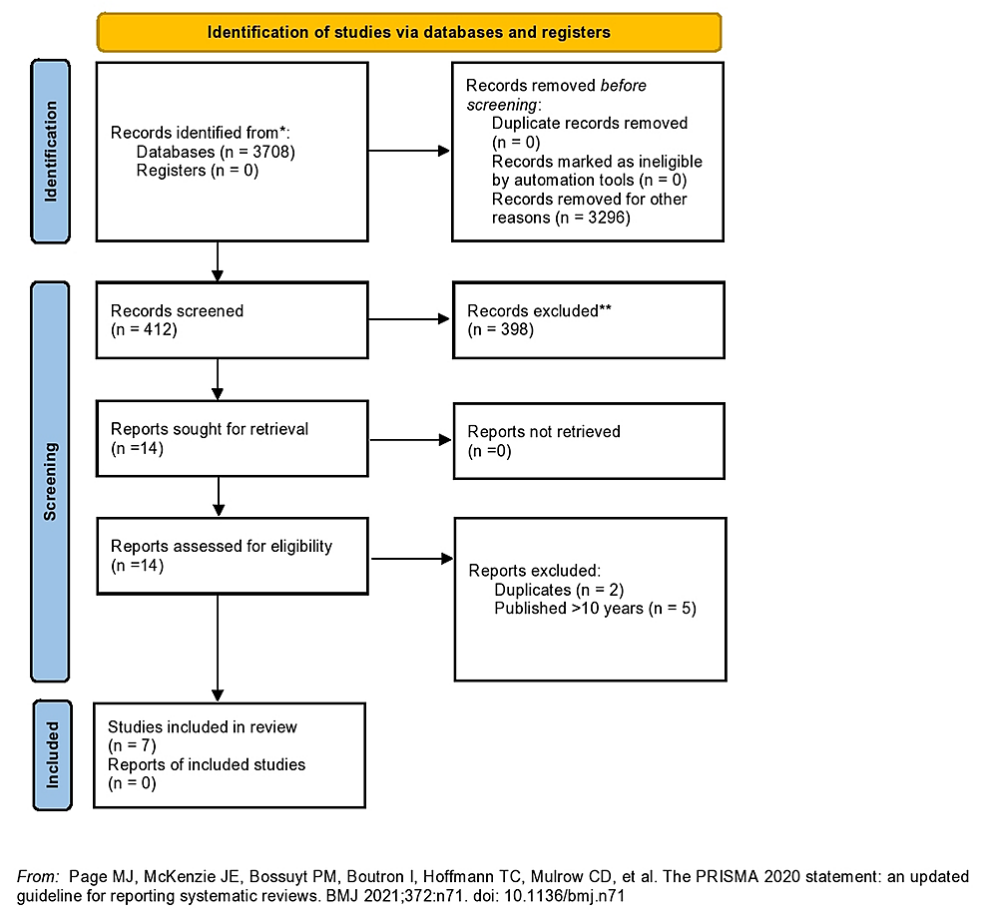
PRISMA 2020 flow diagram for systematic reviews

**Figure 2:**
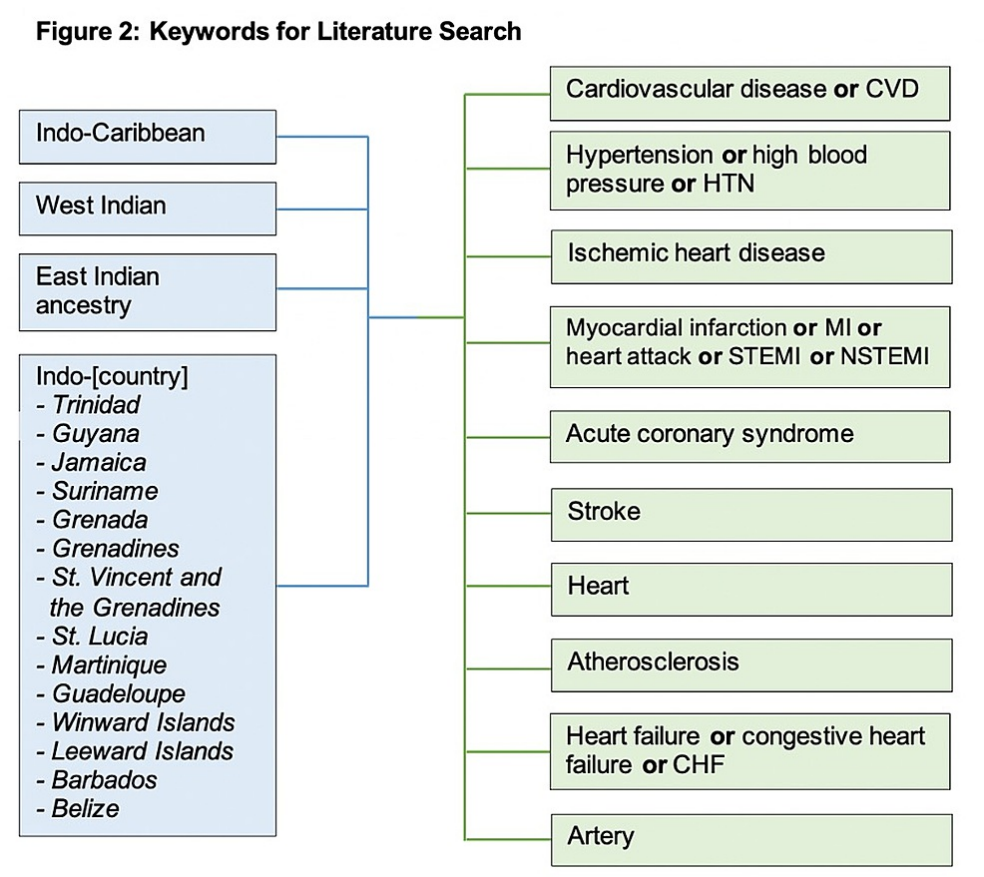
Keywords included in literature search

In order to identify and broadly assess the existing literature about CVD in the Caribbean Community (CARICOM) nations, a thorough scoping review was conducted to synthesize relevant health data. Using the guidance of the 2020 PRISMA systematic review guidelines [20], various health databases including PubMed, PubMed Central, Ovid Medline, LILACS, Medline ProQuest, MedCarib, and EBSCO were queried (Figure 1) using keywords including CVD, HTN, and IHD and specific Indo-Caribbean populations, such as Indo-Trinidad and Indo-Guyana (Figure 2). The specific Caribbean nations that were included in the original search are CARICOM nations in which at least 1% of the total population are of Indian/East Asian descent. Inclusion criteria consisted of relevant articles that described CVD in Indo-Caribbean populations, articles published within the past 10 years, English language, free full text, and peer-reviewed publications. Articles were specifically identified within the last 10 years to ensure the inclusion of up to date information, considering that CVD is highly researched and accumulates new findings rapidly. Free full text publications were specified to highlight the availability of data to the general public, and peer-reviewed publications were included to ensure validity of the data being assessed. Exclusion criteria included publications that were not relevant to CVD, those that identified CVD as a risk factor rather than primary disease, articles that did not stratify findings by Indo-Caribbean ethnicity, and case reports.

The initial search yielded 3708 articles, and 3,296 articles were excluded based on title and abstract review that determined relevancy to the topic, stratification of ethnicity to include Indo-Caribbean populations, and case reports. Out of the 412 articles that were included considering exclusion criteria, these were further scrutinized to ensure that the inclusion criteria were met and that duplicate publications were excluded. Finally, the remaining 14 articles underwent data extraction to further ensure that they were meeting inclusion and exclusion criteria, specifically that the documents were discussing CVD as the primary disease and that the data collected in these studies were stratifying the results based on Indo-Caribbean ethnicity. After this thorough search, this process yielded seven studies that met all inclusion and exclusion criteria.

Prior to conducting the literature search, this review hoped to yield results that detailed various metrics about CVD in the Indo-Caribbean population that could have been assimilated to reveal trends in relevant epidemiology, risk factors, and social determinants of health. However, the results yielded a wide array of information that, while helpful, did not reveal a holistic picture of CVD in the Indo-Caribbean population. While the initial search yielded well over 3,000 papers, less than 1% of papers met inclusion and exclusion criteria - a finding which further illustrated the lack of academic rigor and robust data on this topic. Table 1 lists the studies yielded from the literature review and summarizes important findings from each article.

**Table 1:** Summary of results from publications included in scoping review AMI - acute myocardial infarction; CVD - cardiovascular disease; DM - diabetes mellitus; DPA - dorsalis pedis artery; HPR - high-on treatment platelet reactivity; hs-CRP - high sensitivity C-reactive protein; HTN - hypertension; MS - metabolic syndrome; MPA - medial plantar artery; NT-pro-BNP - N terminal-pro-B-type natriuretic peptide; NSTEMI - non-ST segment elevation myocardial infarction; PCI - percutaneous intervention; PFA - profunda femoris artery; PTA - posterior tibial artery; QOL - quality of life

Publication	Disease entity	Results
Bahall et al., 2019 [21]	AMI	No treatments showed any differences in terms of sex, age, or ethnicity. Of the patients admitted for treatment of an AMI, troponin levels were tested on only 67% of patients and PCI and angioplasty were not offered. Furthermore, 34.4% of patients were current smokers and 31% reported alcohol use. However, little to no patients were given lifestyle modification advice at the time of discharge. Overall, treatment for AMI was centered around pharmacological treatment and referrals, with little inclusion of surgical intervention or lifestyle counseling.
Chadee et al., 2013 [22]	HTN, heart disease	14,793 interview responses were used in this study, with 49.6% identified as Indo-Trinidadian, 35.5% identified as Afro-Trinidadian, and 14.9% identified as mixed. The greatest risk for CVD and its risk factors were found in Indo-Trinidadians and among people who completed only primary school. Furthermore, primary education alone was the greatest risk factor to develop HTN, DM, or CVD. The prevalence of these conditions also increased with age. Additionally, the peak prevalence for HTN was found in the 51-60 year old group.
Ramdass et al., 2014 [23]	Atherosclerosis	Out of 100 patients, 45 were of East Indian descent, 36 of Afro-Caribbean descent, 14 of mixed descent and five had other backgrounds. There were 32 smokers and 69 diabetics. There was a statistically significant difference between East Indians and Afro-Caribbeans with regard to the PFA, PTA, DPA, and MPA with the East Indians having worse disease in the PFA and Afro-Caribbeans having worse disease in the PTA, DPA, and MPA. This portrays that environmental factors such as smoking and diet play a significant role in the disease process rather than solely genetics.
Nayak et al., 2015 [24]	CVD	NT-pro-BNP levels were elevated along with systolic blood pressure, triglycerides, and glucose within the East Indian sub-population while only the systolic blood pressure was elevated in the African sub-population. The East Indian sub-group fulfilled the criteria for MS and, therefore, had a higher risk for CVD within the Trinidad population. As a result, the hs-CRP and NT-pro-BNP levels can be deemed a sufficient marker for MS in high-risk subgroups for CVD in the Trinidad population.
Bahall & Khan, 2018 [25]	AMI	81.2% of the participants were Indo-Trinidadian. A lower QOL was found with women and patients with NSTEMI, DM, HTN and renal disease, and ischemic heart disease. Lack of exercise and stress contributed to a lower QOL while consuming alcohol and eating out were associated with a higher QOL. No associations were found between QOL and ethnicity, hypercholesterolemia, and smoking. QOL in patients with AMI improves over time and will be enhanced earlier with cardiac rehabilitation and psychological support.
Hosein, et al., 2020 [26]	CVD	Cardiovascular disease risk prediction models (ASSIGN, Framingham, and QRISK) underestimated the risk for individuals with CVD up 2.5 times more than they overestimated the risk for healthy individuals. Less than 62% of the CVD cases were identified. As a result, these risk prediction models should be used with discretion on a Trinidad and Tobago population that is intermediate and high risk for CVD.
Seecheran, et al., 2019 [27]	Clopidogrel resistance	Out of 40 individuals, 28 were Indo-Trinidadian, seven were Afro-Caribbean, and five were mixed. 60.7% of the Indo-Trinidadians had HPR, whereas 14.3% of Afro-Caribbean and 40% of mixed ethnicity had HPR. The prevalence in HPR is significantly higher in Indo-Trinidadians when compared to the other sub-groups.

This scoping review defined CVD to include myocardial infarction, IHD, HTN, cerebrovascular disease, congestive heart failure, and atherosclerosis. The literature search yielded publications that only discussed AMI, HTN, and atherosclerosis. While all studies that were included in the search stratified their included populations by ethnicity, most of these studies did not further stratify by gender. Three out of seven of the papers discussed AMI, two of which included medical management and one assessed quality of life (QOL). The remaining publications assessed CVD risk scores, the relationship of NT-pro-BNP and high-sensitivity C-reactive protein with MS in patients with CVD, arteriosclerotic lesions, and self-reported prevalence of heart disease and HTN in Indo-Caribbean populations.

Notably, no publications included in the literature search included comprehensive social determinants of health variables in the Indo-Caribbean population. Previously conducted research in other races and ethnicities has established the role of barriers of healthcare and other determinants of health in the incidence of various diseases; thus, it is highly concerning that extremely limited research exists regarding social determinants of health in Indo-Caribbeans. The interplay between genetics and environment in the development of disease has been extensively logged, and the absence of research detailing the social and environmental components of CVD further suggests that management and treatment of these diseases are not adequate to meet the health needs of this burgeoning population.

It is important to note that methodological limitations to this review may exist, which can contribute to the bias of the results. For example, various health databases were queried to identify relevant data; however, those selected may not fully encompass the entirety of available information regarding CVD in Indo-Caribbean populations. Nonetheless, the databases that were searched identified multiple duplicate publications, which further enhance the paucity of data available. Additionally, excluding studies that were not free full-text publications may eliminate studies that identified key data in regards to individuals in CARICOM nations. The authors chose to exclude these papers because it further highlights the inaccessibility to the general public to relevant health data that may further guide research into risk stratification models and management algorithms specific to Indo-Caribbeans.

Despite the methodological limitations of this review, it is evident that there is an enormous need for increased effort to be placed on conducting CVD research in the Indo-Caribbean population. This is of urgent importance given the COVID-19 pandemic challenges faced by underserved communities. While there is minimal knowledge and data being collected in regards to risk factors, prevention, prevalence and incidence, and management of disease in the Indo-Caribbean population, the fear of developing CVD is pervasive. Community health needs assessment by NYU Langone collected data about CVD risk factors and health behaviors from the Indo-Caribbean community in New York City and found that 65% of the correspondents had a major concern for themselves or their family for developing CVD [5]. The incidence of HTN between Indo-Caribbeans compared to New Yorkers was nearly equivalent; 28% of Indo-Caribbeans and 29% of New Yorkers reported being diagnosed with HTN [5]. However, the difference in the incidence of hyperlipidemia between Indo-Caribbeans and New Yorkers was significant; 41% of Indo-Caribbeans and 30% of New Yorkers reported having hyperlipidemia [5]. Additional health behaviors such as smoking tobacco, consuming alcohol, sleep hygiene, physical activity, and access to healthy foods were assessed, albeit not in consideration to their effects on increasing risk for CVD [5]. These statistics raise concern for increased CVD burden in Indo-Caribbeans compared to the general population, but the data are not being collected. 

The public health implications do not end at the health complications of CVD; rather, substantial economic and governmental impacts continue to raise concern. At the macroeconomic level, the CVD burden in the general population cost the government $555 billion in 2016, and this number is expected to increase astronomically to $1.1 trillion by 2035 [6]. Because of the high prevalence of CVD in the Indo-Caribbean population, it could be extrapolated that this population may play a significant role in the economic burden of CVD in the United States. The exact extent to which the CVD burden in Indo-Caribbeans affects the economy and government is yet to be determined, which further brings to attention issues in the system that may contribute to the high disease burden in this population. For example, the U.S. Census does not list Indo-Caribbean as an ethnicity, and statewide health databases such as Florida Charts do not stratify health data based on ethnicity altogether. In addition, the complex ethnic heterogeneity of the Caribbean community and its effects on the manifestation of preventable diseases should be further studied. In a state where a huge proportion of the Indo-Caribbean population is located, this is especially problematic and poses extreme difficulty in studying the population. Without this vital epidemiologic information, changes cannot be tailored for the prevention, management and treatment of CVD. 

There are many options the authors suggest to improve the collection of quality health data in addition to improving management and treatment options. First and foremost, we call for a change in data collection across all ethnicities in the U.S. Census, specifically adding more diverse ethnicities to include the Indo-Caribbean population. Furthermore, health databases should be required to include ethnicity in the data they collect. The diverse makeup of the United States renders race stratification without the inclusion of ethnicity an obstacle to understanding the complexity of disease manifestation.

Additionally, robust and quality data could allow researchers to develop a risk stratification model that may guide the prevention and treatment of CVD in the Indo-Caribbean population. This option allows for a proactive approach rather than the existing reactive response. More studies that assess risk factors, both modifiable and non-modifiable, are required. Current risk stratification models include ASSIGN, Framingham, and QRISK2, all of which use risk factors to determine the percent risk of experiencing a CVD event within the next 10 years [26]. However, these models have performed poorly in Caribbean countries and minority populations, given that they were not designed with such population’s cultural and social characteristics in mind [26]. As a result, there have been instances of misclassification of individuals who do and do not require CVD treatment, rendering the usage of these models almost counterproductive in certain populations such as Indo-Caribbeans [26]. Future studies in this area may assess the possibility of creating a new or modifying an existing model that includes specific factors that affect cardiovascular health in this population such as genetics, diet, cultural factors, and prevalence of comorbidities.

## Conclusions

The literature search that initiated this review began with over 3,700 articles. Once reviewed, only seven remained. While research is well intentioned to uncover epidemiologic and other public health patterns, this objective cannot be achieved when large groups of people go unseen in aggregated data. Expanding options for demographic data collection can begin to mitigate this issue. Including Indo-Caribbeans in more health research can help to inform interventions, increase health agency among this population, and overall address relevant, preventable health issues.

## Appendices

**Table 2:** Abbreviations in alphabetical order

Abbreviation	Meaning
AMI	Acute myocardial infarction
CARICOM	Caribbean Community
CVD	Cardiovascular disease
DM	Diabetes mellitus
DPA	Dorsalis pedis artery
IHD	Ischemic heart disease
HPR	High on-treatment platelet reactivity
hs-CRP	High sensitivity C-reactive protein
HTN	Hypertension
LMIC	Lower and middle income countries
MPA	Medial plantar artery
MS	Metabolic Syndrome
NCD	Noncommunicable disease
NT-pro-BNP	N-terminal-pro-B-type natriuretic peptide
RHD	Rheumatic heart disease
PCI	Percutaneous intervention
PFA	Profunda femoris artery
PTA	Posterior tibial artery
QOL	Quality of life
